# Diagnostic Challenges and Surgical Management of Second Branchial Arch Anomalies in Adult Patients

**DOI:** 10.7759/cureus.117728

**Published:** 2026-10-10

**Authors:** Samuel J Arthur, Christopher Philus, Emmanuel Lazarus, Pranay Gaikwad, Cecil T Thomas

**Affiliations:** 1 General Surgery, Christian Medical College Vellore, Vellore, IND; 2 Surgery, Christian Medical College Vellore, Vellore, IND

**Keywords:** bailey classification, branchial cleft cyst, neck swelling, recurrence, second branchial arch anomaly

## Abstract

Background

Second branchial cleft anomalies (SBCAs) are the most prevalent anomalies of the branchial apparatus. Their presentation in adults poses a diagnostic challenge due to their resemblance to other cystic neck pathologies and is infrequently reported.

Materials and methods

This retrospective case series reviewed 52 adult patients who underwent surgical treatment for SBCA at a tertiary center over a 15-year period (January 2009 to December 2023).

Results

Branchial cysts were the most common presentation, accounting for 35 patients (67.3%), typically presenting as right-sided upper neck swelling, and were more common in women (30 patients, 57.7%). Preoperative imaging was performed in 44 patients (84.6%), with CT and MRI demonstrating diagnostic accuracies of 86.7% and 84%, respectively. Preoperative and postoperative diagnoses were concordant in 41 patients (80.4%); most discordance arose from lesions suspected to be tuberculous lymphadenitis or malignant neck nodes. Surgical excision was performed in 51 patients (98.1%), with postoperative complications occurring in six patients (11.5%), predominantly nerve palsies. Follow-up data were available for 28 patients (53.8%). Recurrence occurred in two patients (7.1%), both of whom had undergone prior procedures before definitive surgery and had Bailey type III lesions.

Conclusions

Successful management requires a high index of clinical suspicion supported by comprehensive imaging for accurate diagnosis in adults. Complete surgical excision by an experienced surgeon achieves a low recurrence rate, although prior interventions and nerve-related morbidity remain relevant risks requiring careful counseling.

## Introduction

Second branchial cleft anomalies (SBCAs) account for approximately 90-95% of branchial cleft anomalies and, although commonly congenital, often persist into or first present in adulthood during the third to fourth decades [1]. Incomplete obliteration of the branchial apparatus during embryogenesis is the most recognized theory underlying the formation of these anomalies, and SBCAs can occur anywhere from the skin of the supraclavicular fossa to the pharynx at the level of the tonsillar fossa [2]. Cystic neck lesions in adults should be considered cystic metastases until proven otherwise, thus posing a diagnostic challenge. In regions where tuberculous cervical lymphadenitis is common, adult neck swellings are frequently investigated for this condition, which may delay the diagnosis of an underlying branchial anomaly [3]. Misdiagnosis and prior intervention for a presumed alternative diagnosis can distort the anatomy and predispose patients to recurrence after definitive surgery. We present our institutional experience with adult SBCAs, describing clinical presentation, diagnostic accuracy, and surgical outcomes, including factors associated with recurrence.

## Materials and methods

This retrospective case series included 52 adult patients (aged older than 17 years) who underwent surgical treatment for SBCA at our institution between January 2009 and December 2023. Patients were identified from our surgical database after approval from the Institutional Review Board (Silver Ethics and Research Committee) (approval 2508147). Individual informed consent was waived due to the retrospective nature of this analysis, and written consent for surgical and clinical photography had been obtained from all patients at the time of treatment. Diagnosis was based on history, clinical examination, imaging, and intraoperative findings and was confirmed by histopathological examination. Patients younger than 17 years, those managed nonoperatively, and those with histopathology suggestive of other pathologies were excluded.

Data recorded included age, sex, side and duration of symptoms, prior procedures, imaging modality used, preoperative working diagnosis, Bailey classification on imaging and at final assessment, type of surgery and incision, intraoperative tract findings, histopathological diagnosis, and postoperative complications. Preoperative diagnoses were compared with the final diagnosis and categorized as concordant or discordant. A diagnosis was considered concordant when the preoperative and final diagnoses confirmed a second branchial arch anomaly or when a second branchial arch anomaly was included in the preoperative differential diagnosis. Diagnostic accuracy of imaging was based on the imaging report confirming the presence or absence of a second branchial arch anomaly. Bailey type was determined preoperatively from the imaging report and intraoperatively from the operative findings. Recurrence and postoperative morbidity were assessed from outpatient records. Patients without a documented postoperative visit were classified as lost to follow-up, and their recurrence status was considered unknown rather than assumed to be recurrence-free.

## Results

The mean age at presentation was 28.75 years (range, 17-56 years); 30 patients (57.7%) were women. Branchial cysts were the most common lesions (35 patients, 67.3%; mean age, 31.5 years), followed by sinuses or fistulae (17 patients, 32.7%; mean age, 23.2 years). This difference is consistent with the more frequent congenital or childhood onset of symptoms associated with sinuses and fistulae. Lesions were predominantly right-sided (30 patients, 57.7%), with left-sided involvement in 21 patients (40.4%) and bilateral involvement in one patient (1.9%). Nineteen patients (36.5%) had undergone a prior procedure (aspiration, incision and drainage, or excision) before presentation.

Preoperative imaging was performed in 44 patients (84.6%). CT and MRI showed diagnostic accuracies of 86.7% and 84%, respectively. Bailey classification on imaging showed predominantly type II lesions (33 patients, 63.5%), followed by type III (12 patients, 23.1%) and type I (seven patients, 13.5%). The Bailey classification assigned on imaging matched the final intraoperative classification in 81.4% of evaluable patients (35/43). Fine-needle aspiration cytology was performed in only 10 patients; an adequate sample was obtained in seven. All seven samples were suggestive of a benign cystic lesion, with branchial cyst considered a possibility in four patients. Preoperative and postoperative diagnoses were concordant in 80.4% of evaluable patients (41/51). Discordant cases almost always involved lesions initially suspected to represent tuberculous lymphadenitis or malignant cervical lymphadenopathy (Table 1).

**Table 1:** Demographics, clinical presentation, and diagnostic workup of patients with second branchial arch anomalies (n = 52). One patient was excluded from the concordance analysis owing to a missing preoperative working diagnosis; nine patients were excluded from the Bailey-type matching analysis owing to absent or unrecorded imaging classification.

Characteristic	Cyst (n = 35)	Sinus/fistula (n = 17)	Total, n (%)
Age at presentation (years), mean	31.5	23.2	28.75 (SD, 10.6)
Sex, women:men	18:17	12:05	30:22 (57.7:42.3)
Side of lesion			
Right	18	12	30 (57.7)
Left	17	4	21 (40.4)
Bilateral	0	1	1 (1.9)
Prior procedure before presentation			
History of prior procedures	14	5	19 (36.5)
No prior procedures	21	12	33 (63.5)
Fine-needle aspiration cytology performed	10	0	10 (19.2)
Imaging modality used			
Ultrasonography	13	1	14 (26.9)
CT (including sinusogram)	12	4	16 (30.8)
MRI	6	6	12 (23.1)
Sinusogram alone	0	2	2 (3.8)
No imaging performed	4	4	8 (15.4)
Bailey classification postoperatively			
Type I	6	1	7 (13.5)
Type II	25	8	33 (63.5)
Type III	4	8	12 (23.1)
Preoperative/postoperative diagnostic concordance (n = 51 evaluable)			
Concordant	-	-	41 (80.4)
Discordant	-	-	10 (19.6)
Imaging-based Bailey type matched postoperative type (n = 43 evaluable)	-	-	35 (81.4)

Surgical excision was performed in 51 patients (98.1%). A transverse cervical incision was used in 39 patients, and a stepladder incision was used for more complex tracts in 14 patients. One patient with bilateral involvement required both approaches. One patient with an infected branchial cyst was managed with incision and drainage. A discrete tract was identified intraoperatively in 22 patients, reaching the pharyngeal wall in 15 patients. The median hospital stay was 3.3 days.

Postoperative complications occurred in six patients (11.5%): four nerve palsies, including two hypoglossal nerve palsies, one of which was permanent, and two cases of marginal mandibular nerve weakness; one surgical site infection; and one seroma. Documented postoperative follow-up was available for 28 patients (53.8%); the remaining 24 had no recorded outpatient visit and were classified as lost to follow-up (Table 2). Among the 28 patients with follow-up, recurrence occurred in two (7.1%). Both had Bailey type III lesions and a history of prior procedures before definitive surgery. Both were managed successfully by re-excision (Table 3).

**Table 2:** Surgical management and postoperative outcomes of patients with second branchial arch anomalies (n = 52). One patient with bilateral involvement is counted under both incision types; therefore, the total number of incisions exceeds 52. Both recurrences occurred in patients with Bailey type III lesions and a history of prior procedures before definitive surgery: one patient in the cyst group and one in the sinus/fistula group.

Characteristic	Cyst	Sinus/fistula	Total, n (%)
Type of surgery			
Excision	34	17	51 (98.1)
Incision and drainage only	1	0	1 (1.9)
Incision used			
Transverse cervical	32	7	39 (75.0)
Stepladder	3	11	14 (26.9)
Postoperative complications (n = 6, 11.5%)			
Nerve-related	3	1	4 (7.7)
Seroma	1	0	1 (1.9)
Surgical site infection	1	0	1 (1.9)
Follow-up status (n = 52)			
No recurrence	21	5	26 (50.0)
Recurrence	1	1	2 (3.8)
Lost to follow-up	13	11	24 (46.2)

**Table 3:** Clinical data of the two patients with recurrence.

Serial no.	Age at surgery (years)	Sex	Side affected	Previous procedures	Diagnosis at index surgery	Imaging	Surgery performed	Bailey type of lesion	Postoperative biopsy	Time to recurrence	Re-excision	Incision	Postoperative biopsy findings	Lining epithelium	Follow-up	Re-recurrence
1	31	Male	Left	One (excision)	Recurrent second branchial cyst	Ultrasound	Cyst excision	Type III	Branchial cyst	One month	Yes	Transverse	Recurrent branchial cyst	Stratified squamous	180 months	No
2	18	Female	Left	Three (incision and drainage once, excision twice)	Recurrent branchial sinus	MRI	Sinus excision	Type III	Branchial cleft sinus	Five months	Yes	Stepladder	Recurrent branchial sinus	Stratified squamous	10 months	No

## Discussion

Adult second branchial arch anomalies are uncommon and are often misdiagnosed as other cystic neck pathologies at presentation, particularly tuberculous lymphadenitis and metastatic nodal disease [4]. Preoperative diagnostic accuracy for branchial arch anomalies is sparsely reported in the literature. The few available series suggest that concordance rates have increased from around 52% a decade ago to 85-90% more recently, although most studies combined pediatric and adult cohorts [5-7]. Our study is the first restricted to adult patients and showed 80.4% concordance between preoperative and final diagnoses. This finding reinforces the need for a high degree of suspicion for branchial anomalies, even when the initial clinical diagnosis favors an infectious or malignant cause. Cross-sectional imaging helps improve preoperative diagnostic accuracy [8,9] by providing information about the anatomical relationships between the cyst or sinus and surrounding structures, but it has been shown to be inferior to CT fistulography in delineating sinus or fistula tracts [10]. In this series, CT and MRI showed diagnostic accuracies of 86.7% and 84%, respectively. However, modality assignment was not randomized; therefore, selection bias limits these findings to observational comparisons within this series. The Bailey classification [11], proposed in 1929, classifies SBCAs into four types (Figure 1) based on their location and relationship to anatomical structures, which is helpful in surgical planning.

**Figure 1:**
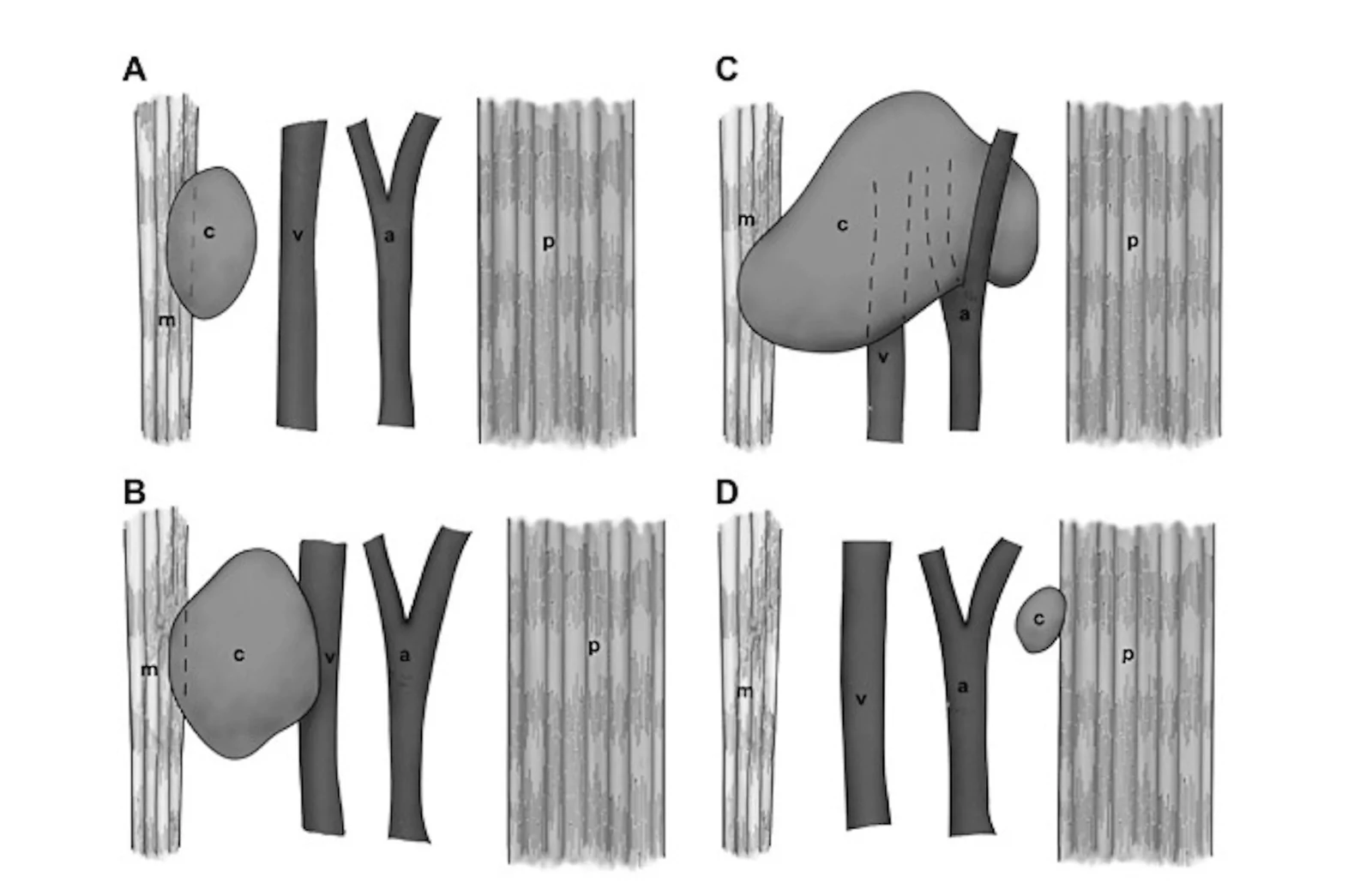
Bailey’s classification of SBCAs. (A) Type I: The cyst (c) is superficial to the anterior border of the sternocleidomastoid muscle (m). (B) Type II: The cyst (c) is adjacent to the carotid sheath. (C) Type III: The cyst (c) passes between the internal and external carotid arteries and extends to the lateral wall of the pharynx (p). (D) Type IV: The cyst is deep to the carotid sheath and abuts the pharynx. SBCAs, second branchial cleft anomalies Reprinted from Neuroimaging Clinics of North America, vol. 10, Mukherji et al. [12], Imaging of congenital anomalies of the branchial apparatus, pp. 75-93, Copyright © 2000 Elsevier, with permission.

Our cohort included 19 patients (36.5%) who presented with recurrent neck swellings and a history of previous procedures, such as cyst aspiration or drainage. This finding is consistent with the available literature suggesting that aspiration of neck cysts and drainage of infected branchial abscesses are associated with a higher risk of recurrence [13].

Complete surgical excision, most often via a transverse cervical incision, remained the mainstay of treatment in this series, consistent with the broader surgical literature on branchial cyst management [14]. The stepladder-pattern incision, shown in Figure 2 and first described by Bailey in 1933, is used for complex cases, especially branchial fistulas or sinuses [11]. Intraoperatively, only 15 patients (28.8%) had a tract extending to the pharyngeal wall, supporting established observations that complete fistulas are rare in second branchial arch anomalies [15]. Recurrence occurred in two patients (7.1%) with documented follow-up (28/52). Both had undergone a prior procedure before definitive surgery, and both had Bailey type III lesions, whose deep course between the internal and external carotid arteries may make complete excision technically more challenging. These observations are consistent with the literature, reinforcing that prior infection and procedures are major risk factors for recurrence because of residual tissue and scarring of surgical planes [16,17]. However, with only two recurrence events and a small sample size, these findings remain observational and preclude formal statistical analysis.

**Figure 2:**
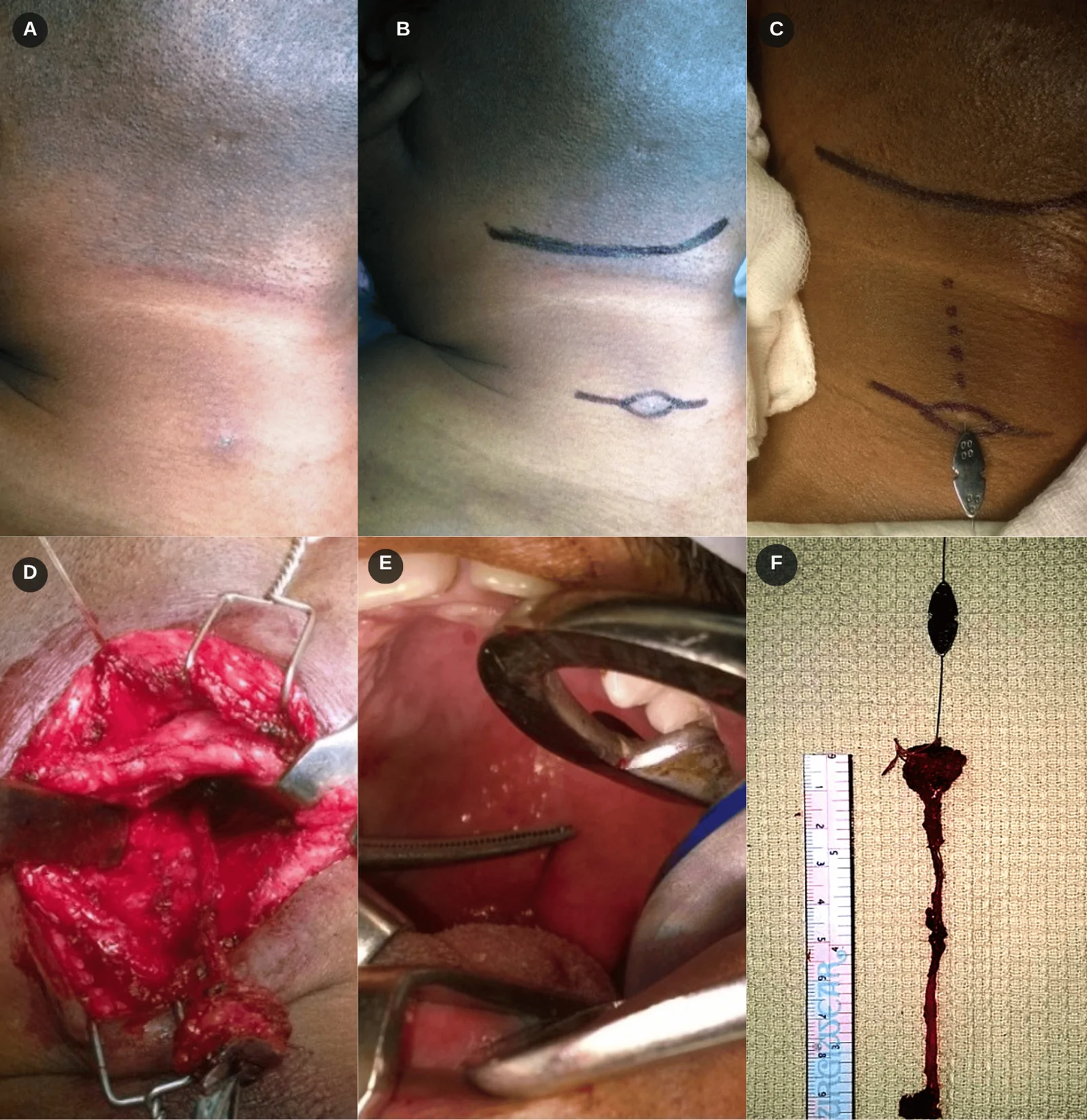
Stepladder incision for excision of a branchial fistula. (A) Preoperative image of the branchial fistula. (B) Incisions marked around the external opening and at the level of the carotid bifurcation. (C) A lacrimal probe used to delineate the tract, aiding its identification during dissection. (D) Course of the fistula tract below the mandible. (E) Internal opening identified in the oropharynx. (F) Specimen of the branchial fistula after excision.

Nerve-related complications occurred in four patients (7.7%), making them the most common morbidity in this series. Three of these patients had a history of prior interventions. These findings suggest a possible association between prior interventions and postoperative morbidity, although the small sample size precludes definitive conclusions. These complications reflect the proximity of second branchial arch lesions and their associated tracts to the hypoglossal nerve and the marginal mandibular branch of the facial nerve. This underscores the technical demands of dissection in this region, particularly in reoperative or heavily scarred surgical fields. Careful preoperative counseling regarding this risk is warranted, particularly in patients with a history of prior interventions. None of our patients underwent ipsilateral tonsillectomy, consistent with published data indicating that ipsilateral tonsillectomy does not affect recurrence rates [18].

This series has important limitations. Documented follow-up was available for only 28 of 52 patients, so the observed recurrence rate is a minimum estimate rather than the true cohort-wide rate and should be interpreted with caution. With only two recurrence events, formal statistical analysis of risk factors was not possible, limiting our findings to hypothesis-generating associations rather than established risk factors. The retrospective, single-center design is also subject to selection and recall bias, and there was no standardized follow-up protocol across the 15-year study period.

## Conclusions

Adult SBCAs are frequently mistaken for tuberculous lymph node disease or malignancy and require a high index of clinical suspicion, supported by comprehensive imaging, to avoid diagnostic delay. Complete excision by an experienced head and neck surgeon offers good outcomes with low morbidity. Although the observed associations between recurrence, prior interventions, and Bailey type III lesions remain exploratory, larger prospective cohorts with long-term follow-up are needed to establish definitive risk factors for recurrence in adults.

## References

[1] Prescher H, Nathan SL, Bauer BS, Reid RR (2022). Branchial cleft anomalies: embryogenesis, clinical features, diagnosis, and management. FACE.

[2] Prosser JD, Myer CM 3rd (2015). Branchial cleft anomalies and thymic cysts. Otolaryngol Clin North Am.

[3] Guldfred LA, Philipsen BB, Siim C (2012). Branchial cleft anomalies: accuracy of pre-operative diagnosis, clinical presentation and management. J Laryngol Otol.

[4] Gourin CG, Johnson JT (2000). Incidence of unsuspected metastases in lateral cervical cysts. Laryngoscope.

[5] Kenealy JF, Torsiglieri AJ Jr, Tom LW (1990). Branchial cleft anomalies: a five-year retrospective review. Trans Pa Acad Ophthalmol Otolaryngol.

[6] Schroeder JW Jr, Mohyuddin N, Maddalozzo J (2007). Branchial anomalies in the pediatric population. Otolaryngol Head Neck Surg.

[7] Daoud FS (2005). Branchial cyst: an often forgotten diagnosis. Asian J Surg.

[8] Wang HK, Tiu CM, Chou YH, Chang CY (2003). Imaging studies of pyriform sinus fistula. Pediatr Radiol.

[9] Adams A, Mankad K, Offiah C, Childs L (2016). Branchial cleft anomalies: a pictorial review of embryological development and spectrum of imaging findings. Insights Imaging.

[10] Ryu CW, Lee JH, Lee HK, Lee DH, Choi CG, Kim SJ (2006). Clinical usefulness of multidetector CT fistulography of branchial cleft fistula. Clin Imaging.

[11] Bailey H (1929). Branchial cysts, and other essays on surgical subjects in the facio-cervical region. Br J Surg.

[12] Mukherji SK, Fatterpekar G, Castillo M, Stone JA, Chung CJ (2000). Imaging of congenital anomalies of the branchial apparatus. Neuroimaging Clin N Am.

[13] Al-Mufarrej F, Stoddard D, Bite U (2017). Branchial arch anomalies: recurrence, malignant degeneration and operative complications. Int J Pediatr Otorhinolaryngol.

[14] Bajaj Y, Ifeacho S, Tweedie D (2011). Branchial anomalies in children. Int J Pediatr Otorhinolaryngol.

[15] Meijers S, Meijers R, van der Veen E, van den Aardweg M, Bruijnzeel H (2022). A systematic literature review to compare clinical outcomes of different surgical techniques for second branchial cyst removal. Ann Otol Rhinol Laryngol.

[16] Prasad SC, Azeez A, Thada ND, Rao P, Bacciu A, Prasad KC (2014). Branchial anomalies: diagnosis and management. Int J Otolaryngol.

[17] Anand TS, Tatavarty S, Pal S, Chabra E, Kumar S (2007). Oropharyngeal second branchial cleft cyst. Int J Pediatr Otorhinolaryngol Extra.

[18] Cheng J, Elden L (2012). Management of pediatric second branchial fistulae: is tonsillectomy necessary?. Int J Pediatr Otorhinolaryngol.

